# Designed α-sheet peptides suppress amyloid formation in *Staphylococcus aureus* biofilms

**DOI:** 10.1038/s41522-017-0025-2

**Published:** 2017-07-03

**Authors:** Alissa Bleem, Robyn Francisco, James D. Bryers, Valerie Daggett

**Affiliations:** Department of Bioengineering, University of Washington, Seattle, WA 98195-5013 USA

## Abstract

Nosocomial infections affect hundreds of millions of patients worldwide each year, and ~60% of these infections are associated with biofilm formation on an implanted medical device. Biofilms are dense communities of microorganisms in which cells associate with surfaces and each other using a self-produced extracellular matrix composed of proteins, polysaccharides, and genetic material. Proteins in the extracellular matrix take on a variety of forms, but here we focus on functional amyloid structures. Amyloids have long been associated with protein misfolding and neurodegenerative diseases, but recent research has demonstrated that numerous bacterial species utilize the amyloid fold to fortify the biofilm matrix and resist disassembly. Consequently, these functional amyloids, in particular the soluble oligomeric intermediates formed during amyloidogenesis, represent targets to destabilize the extracellular matrix and interrupt biofilm formation. Our previous studies suggested that these amyloidogenic intermediates adopt a non-standard structure, termed “α-sheet”, as they aggregate into soluble oligomeric species. This led to the design of complementary α-sheet peptides as anti-α-sheet inhibitors; these designs inhibit amyloidogenesis in three unrelated mammalian disease-associated systems through preferential binding of soluble oligomers. Here we show that these anti-α-sheet peptides inhibit amyloid formation in *Staphylococcus aureus* biofilms. Furthermore, they inhibit aggregation of pure, synthetic phenol soluble modulin α1, a major component of *Staphylococcus aureus* functional amyloids. As it aggregates phenol soluble modulin α1 adopts α-helix then α-sheet and finally forms β-sheet fibrils. The binding of the designed peptide inhibitors coincides with the formation of α-sheet.

## Amyloid proteins: Peptides prevent folding

Small protein fragments called peptides can be designed to interfere with the folding of proteins in biofilms and disrupt biofilm formation. Valerie Daggett and colleagues at the University of Washington in Seattle, USA, focused their attention on a type of folded proteins called amyloids. Some amyloid proteins are well known for their role in neurodegenerative diseases, but others can protect biofilms from degradation, making them resistant to treatment or to removal from medical devices. The researchers made peptide molecules, composed of linked amino acids, that were designed to physically interfere with the folding processes that create amyloids in biofilms. These peptides were shown to inhibit amyloid formation in biofilms produced by *Staphylococcus aureus* bacteria, which cause many clinically significant infections. The results may offer a novel approach to combat biofilm formation.

## Introduction

Nosocomial infections, or healthcare-associated infections, are the most common adverse event in healthcare delivery worldwide, leading to significant mortality and financial losses in a variety of settings. In the United States in 2014, approximately one in 25 patients contracted at least one infection during the course of hospitalization, and the frequency of hospital-acquired infections in developing countries is expected to be at least three times higher than in the United States.^1, 2^ This problem is compounded by the fact that ~60% of these infections are associated with biofilm formation.^3^ Microbial infections occur within surgical wounds, as well as on nearly all implanted medical devices, including prosthetic heart valves, pacemakers, cerebrospinal fluid shunts, urinary and intravascular catheters, ocular prostheses, and intrauterine contraceptive devices.^4^ When microbes dwell on these surfaces within a biofilm, their susceptibility to antibiotics can decrease by a factor of 10–1000. Sub-lethal doses of antibiotics can actually enhance biofilm formation,^5^ and the spread of antibiotic resistance genes is accelerated in biofilm communities, especially when subjected to antibiotic stress.^6, 7^ Additionally, an increasing number of infectious biofilms are formed by multidrug resistant bacteria, and the heterogeneous matrix architecture of biofilms likely supports multiple mechanisms of resistance.^8^ These issues are further exacerbated by an overall decline in antimicrobial drug development. Indeed, nine classes of antibacterial drugs were introduced between 1936 and 1968, but only five new classes have been approved since then.^9^ Therefore, new approaches are needed to address biofilm-associated nosocomial infections.

Methicillin-resistant *Staphylococcus aureus* (MRSA), in particular, is a major cause of nosocomial infections due to its versatility and arsenal of virulence factors.^10, 11^ When *Staphylococcus aureus* forms a biofilm on a medical device or wound, cells associate with surfaces and each other using a self-produced extracellular matrix (EM) composed of proteins, polysaccharides, and genetic material. Proteins in the EM take on a variety of roles, but recently phenol soluble modulins (PSMs) have been identified as key factors with dual functionality for *S. aureus* biofilms. In their soluble monomeric form, they recruit, activate, and lyse human neutrophils, kill competing bacteria, and promote biofilm dissociation.^12–14^ PSMs also self-associate to form amyloid fibrils that fortify the biofilm matrix to better resist disassembly by matrix degrading enzymes and mechanical stress.^15^ Amyloid fibrils are β-sheet-rich structures that form through the aggregation of normally soluble peptides and proteins. Extracellular deposition of these fibrillar aggregates has long been associated with protein misfolding and human neurodegenerative diseases, such as Alzheimer’s Disease, but numerous bacterial species make use of amyloid fibrils as structural scaffolds to stabilize the biofilm.^16–18^ Consequently, these functional amyloids represent a target to interrupt biofilm formation.

**Fig. 1:**
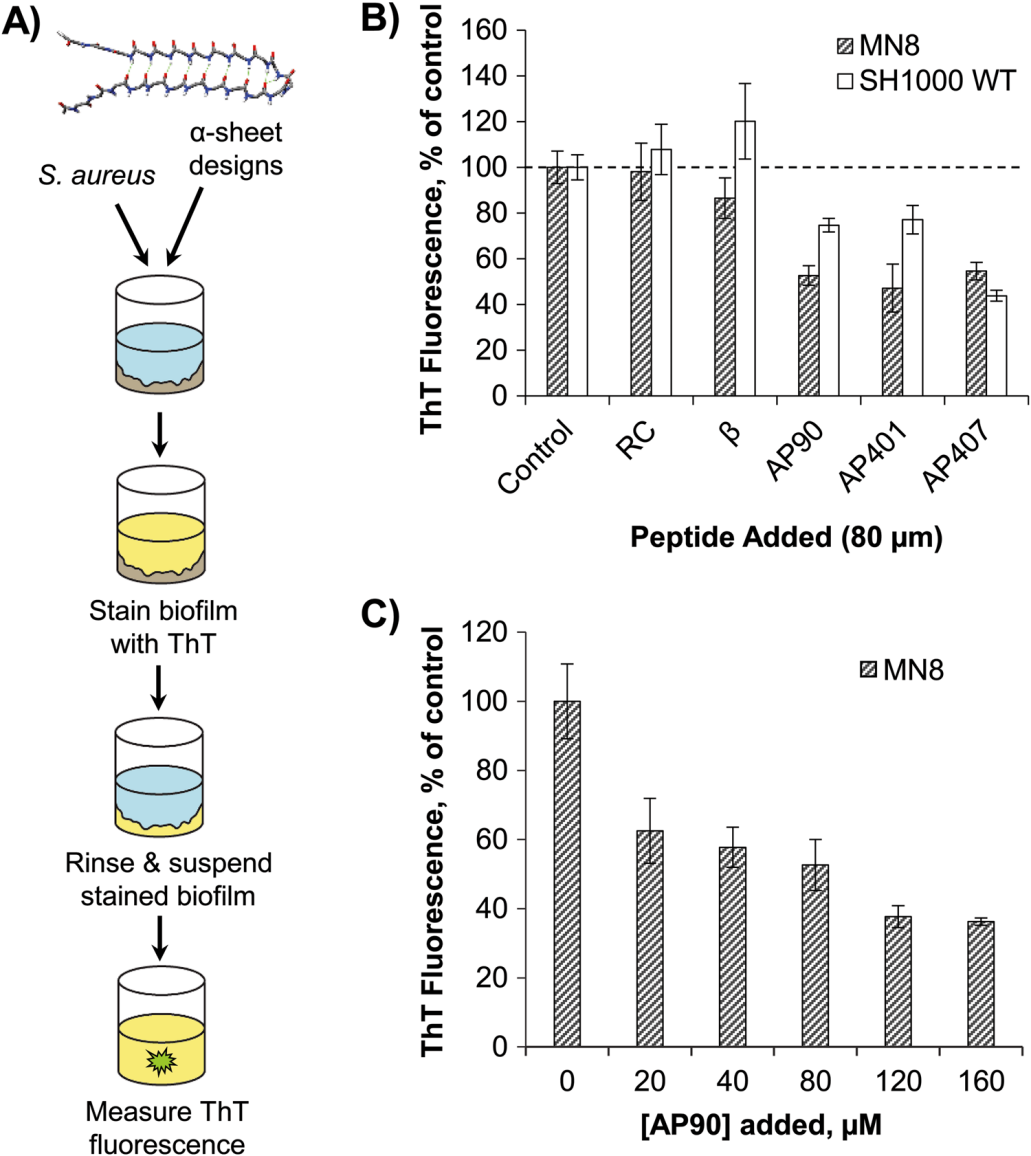
Screening of designed peptides for inhibition of amyloid formation in *S. aureus* biofilms. **a** Schematic of protocol for testing designed α-sheet peptides in *S. aureus* biofilm cultures with an illustration of the main-chain structure of the AP90 α-sheet design. **b** A panel of designed α-sheet peptides (AP90, AP401, and AP407), as well as random coil (RC) and β-hairpin controls (β), was tested against two *S. aureus* strains, MN8 (*hashed bars*) and SH1000 WT (*white bars*). ThT fluorescence fluorescence values indicate the extent of amyloid formation in the EM and are shown as the percent of peptide-free control conditions. **c** Dose-response curve for the designed peptide AP90 against *S. aureus* MN8 biofilms reveals a significant decrease in EM amyloid content as the concentration of AP90 is increased. Error bars in **b** and **c** represent the standard error of the mean for experiments performed in triplicate

The triggers for PSM aggregation are not fully understood, but the conversion of soluble peptides to insoluble fibrils has been extensively characterized for mammalian amyloid systems.^19^ The first step in amyloid fibril formation is a conformational change in the soluble monomers, facilitating their aggregation into oligomeric intermediates, which results in the eventual formation of mature insoluble amyloid fibrils. The amyloid-related toxicity observed in human diseases is linked to the soluble oligomers formed during aggregation, while the mature fibrils are relatively inert.^20^ Structural similarity between soluble oligomers from a range of unrelated proteins has been demonstrated by the binding of the A11 antibody, which is specific for oligomers but does not bind the corresponding starting structures nor amyloid fibrils.^21^ Based on the antibody’s specificity for soluble oligomers with varying amino acid sequences, it was suggested that these toxic oligomers adopt a common but non-standard backbone structure. The amorphous, heterogeneous, and dynamic nature of the oligomeric species has encumbered their structural characterization at high resolution, but we have long been using atomistic molecular dynamics (MD) simulations to map the early conformational changes during amyloidogenesis. In so doing we found a novel secondary structure—“α-sheet”—was populated by a variety of unrelated amyloid proteins and peptides under amyloidogenic conditions.^22, 23^ We hypothesized that this structure is universally adopted during amyloidogenesis and associated with toxicity.^22, 24^ α-sheet is similar to the more conventional β-sheet but it is characterized by the alignment of main chain carbonyl groups on one side of the α-strand and NH groups on the other, as opposed to the alternation of these groups in β-sheets (Fig. 1a). To test our hypothesis we designed peptides in silico to adopt stable, monomeric α-sheet structure (α-sheet hairpins) complementary to the structure observed in our MD simulations.^25^ The best scoring designs were synthesized and tested against three different human disease systems: transthyretin (implicated in systemic amyloidosis), Aβ (linked to Alzheimer’s disease), and amylin (associated with type 2 diabetes) and they inhibited aggregation in all three systems and did so by preferentially binding the toxic soluble oligomers.^25, 26^

If α-sheet is indeed a common intermediate in amyloidogenesis, then it should also be populated during the formation of PSM amyloids in *S. aureus*. Here we test this idea in *S. aureus* biofilm cultures and in vitro with pure, synthetic phenol soluble modulin α1 (PSMα1), one of the main proteinaceous components of *S. aureus* amyloids.^15^ We examined several α-sheet designs for their ability to inhibit PSMα1 fibrillogenesis in abiotic conditions as well as their performance in disrupting biofilm matrix stability in *S. aureus*. The α-sheet peptide designs inhibit amyloid formation in both systems and ultimately compromise the integrity of the *S. aureus* biofilms. Experiments were also performed to shed light on the structural transitions of PSMα1, and our results indicate that the peptide adopts α-sheet structure during amyloid formation. Finally, this is the first study demonstrating the efficacy of these designed peptides outside of a mammalian amyloid system, supporting the generality of the α-sheet hypothesis. Thus, α-sheet inhibitors could prove applicable to a wide variety of amyloidogenic proteins, including functional amyloids produced by other bacterial species such as *Escherichia coli* and *Pseudomonas aeruginosa*.

## Results

We begin with a short description of the α-sheet designs employed here and their spectral properties given the importance of structure in their mechanism of action, as well as what that suggests about the structure of amyloid species with which they interact. In addition, the structural properties of the peptide provide a benchmark, or spectroscopic signature, for α-sheet structure, which can be used to interpret spectra of amyloid species. The main α-sheet design used here is AP90 (Alternating Peptide #90, referred to as *α1* previously^25, 26^), but we also tested several other designs (Supplementary Table S1). α-sheet structure is comprised of successive amino acids alternating between α_R_ and α_L_ conformations, which can be stabilized by using alternating L-amino acids and D-amino acids, as the D-amino acids have inverted conformational propensities thereby leading to higher populations of α_L_, which in turn stabilize the α-sheet.^22–25^ As expected, AP90 does not adopt conventional secondary structure as assessed by circular dichroism (CD), fourier transform infrared spectroscopy, and 2D nuclear magnetic resonance experiments.^25^ Due to the alternating chirality in the strands of the hairpin, there is cancellation of the CD signal, leading to a featureless spectrum, except for negative ellipticity in the so-called random coil region around 195–200 nm due to the L-amino acids in the turn and termini (Supplementary Fig. S1). The AP401 and AP407 designs display similar spectral features by CD, except that AP401 has a positive signal in the random coil region (Supplementary Fig. S1). AP401 has the same sequence as AP90 but with reversed chirality in the strands and turn (Supplementary Table S1), thus the D-amino acids give rise to an approximate mirror image CD spectrum relative to AP90. AP407 is a variant of AP90 with a disulfide bond. AP193 is a more hydrophobic variant of AP90 (with the following changes, l→y, S→F, S→Y, where lower case letters are used for D-amino acids), which also contains a Cys residue for coupling chemistry through the thiol (Supplementary Table S1). An unstructured random coil peptide (RC) and a β-sheet hairpin (β) are used as structural controls.^25, 26^

### Designed α-sheet peptides inhibit amyloid formation in biofilm cultures

We employed two *S. aureus* strains—SH1000 WT, a *rsbU* ^*+*^ laboratory strain,^27^ and MN8, a clinical strain isolated from the human urogenital tract^28^ (Supplementary Table S2). First we confirmed that the cells formed biofilms by batch growth of the bacteria in microtiter plates through staining with Crystal Violet dye after 24 h. Application of our AP90 α-sheet design led to a 54% reduction in biofilm. As crystal violet is a crude measure of the extent of biofilm formation, we assessed amyloid fibril formation in the biofilms by adapting the conventional Thioflavin T (ThT) amyloid fibril assay for use in live cells. After 24 h of batch growth in microtiter plates, the biofilms were washed and incubated with ThT, and the resulting fluorescence signals served as a proxy for the extent of fibril formation in the EM (Fig. 1a). Next, we utilized this assay to test the α-sheet inhibitor designs and control peptides described above (sequences provided in Supplementary Table S1).

To test whether the α-sheet designs inhibit *S. aureus* amyloid formation, 80 μM of the designs and control peptides were added to the growth medium (LB Broth, Lennox) prior to inoculation, corresponding to a starting concentration of 1.51 pg/CFU (picogram per colony forming unit), which dropped exponentially as the cultures were allowed to grow for 24 h at 37 °C. Planktonic cells were removed and the remaining biofilms were assayed for amyloid content using ThT. The resulting fluorescence signals indicated a significant reduction in amyloid fibril formation in the presence of AP90, AP401, and AP407 (Fig. 1b), particularly for the MN8 clinical isolate. For example, the disulfide-linked peptide design, AP407, reduced amyloid fibril formation by 46 and 56% in the MN8 and SH1000 WT strains, respectively. In contrast, the RC and β controls were ineffective. These results suggest that the observed reduction in biofilm amyloid content is due to targeting of α-sheet structure by the designs rather than a nonspecific effect. In addition, fibril formation decreased in a dose-dependent manner when *S. aureus* MN8 biofilms were grown with increasing concentrations of AP90 in the culture medium (Fig. 1c).

### Designed peptide inhibitors disrupt *S. aureus* biofilms

**Fig. 2:**
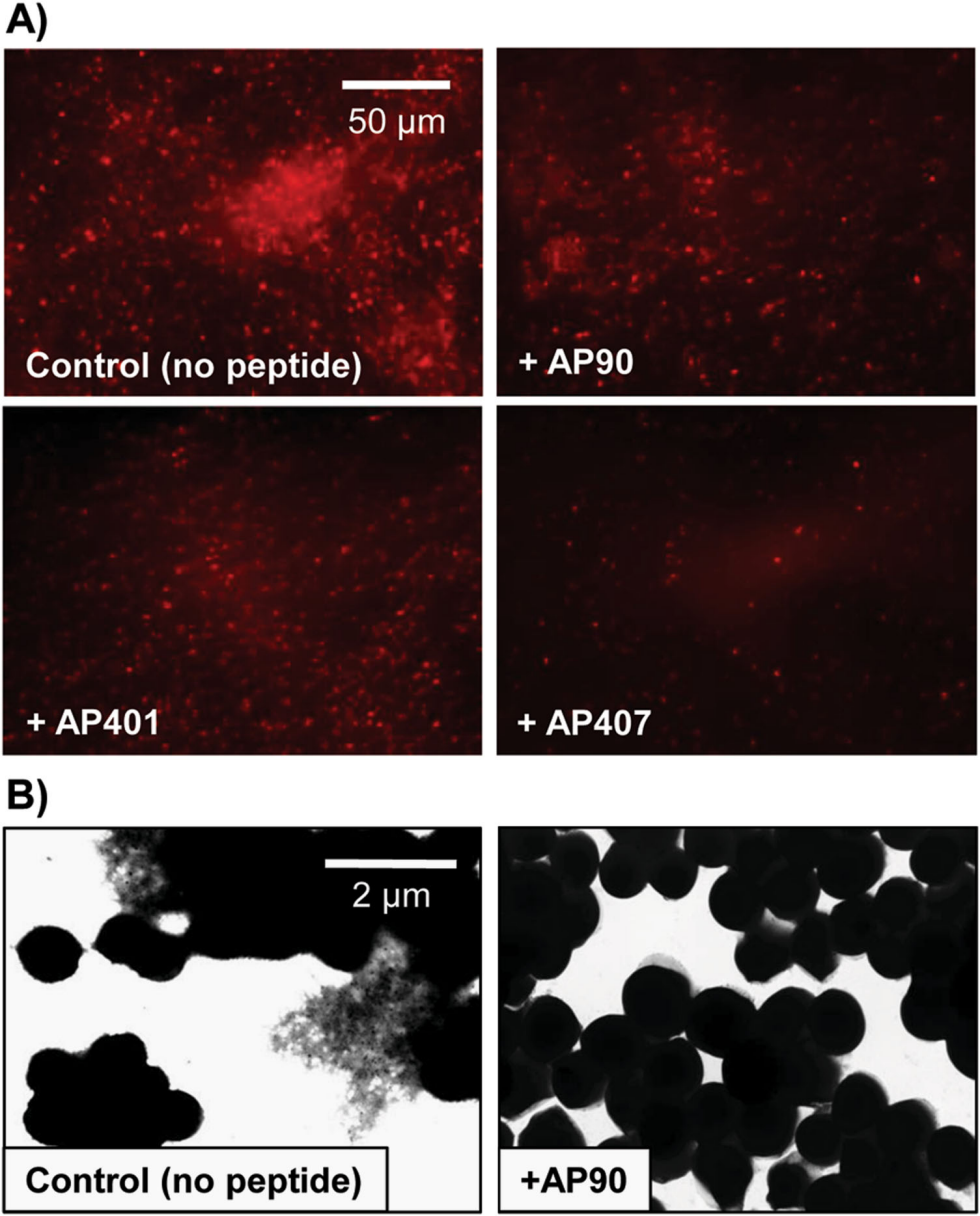
*S. aureus* biofilm structures become less robust when grown in the presence of designed peptide inhibitors. **a** *S. aureus* MN8 + mCherry biofilms were grown on glass substrates for 24 h and then cells were washed and fixed. The peptide inhibitors (80 μM) caused cells to detach during the wash step. Images are representative of triplicate wells. For quantification of the images see Supplementary Fig. S2. **b** In *S. aureus* SH1000 WT biofilms grown in regular LB medium, PSM amyloid fibrils were visible as deposits in spaces between cells (*left*). Upon addition of the designed peptide AP90 (80 μM), no extracellular fibril deposits were observed (*right*)

To further investigate the ability of designed peptides to inhibit PSM amyloid formation, fluorescent (mCherry) *S. aureus* MN8 biofilms were grown in culture plates with glass bottom wells with or without α-sheet inhibitors. After 24 h at 37 °C the biofilms were gently rinsed with pipetted saline to remove unattached biomass and the remaining attached cells were fixed and imaged. A robust biofilm formed on the glass at the bottom of each well in LB medium alone (Fig. 2a); in contrast, there was significant disruption of the biofilm in the presence of the α-sheet compounds. AP407, for example, caused nearly all of the biofilm to detach from the slide upon rinsing. Quantification of the images is presented in Supplementary Fig. S2. These results are in agreement with the results obtained for amyloid inhibition in Fig. 1b. Addition of anti-α-sheet peptides triggered a significant reduction in PSM amyloid formation as measured by ThT fluorescence, which presumably weakened the *S. aureus* biofilms and their attachments by reducing the PSM amyloid fibril content of the EM. To further explore the observed reduction in matrix stability upon treatment with α-sheet peptides, *S. aureus* SH1000 WT biofilms were examined with transmission electron microscopy (TEM). Deposits of PSM fibrils were clearly visible in the spaces around the *S. aureus* cells; however, amyloid fibrils could not be found in the presence of AP90 (Fig. 2b).

### Amyloid formation by PSMα1 is characterized by structural changes

To further characterize the binding of designed α-sheet peptides to PSMs, we first quantified the structural transitions of a synthetic PSM peptide, PSMα1, as it aggregated to form amyloid fibrils in solution. PSMα1 is hydrophobic and requires solubilization with organic solvent to ensure sample homogeneity and removal of any amyloid “seeds” prior to beginning the aggregation reaction. Dimethyl sulfoxide (DMSO) and hexafluoroisopropanol (HFIP) are commonly used for this purpose and past studies of PSMs have used HFIP^15^; however, a potential problem with using HFIP is that it can stabilize α-helical structure. Consequently we investigated the behavior of PSMα1 in both HFIP and DMSO.

**Fig. 3:**
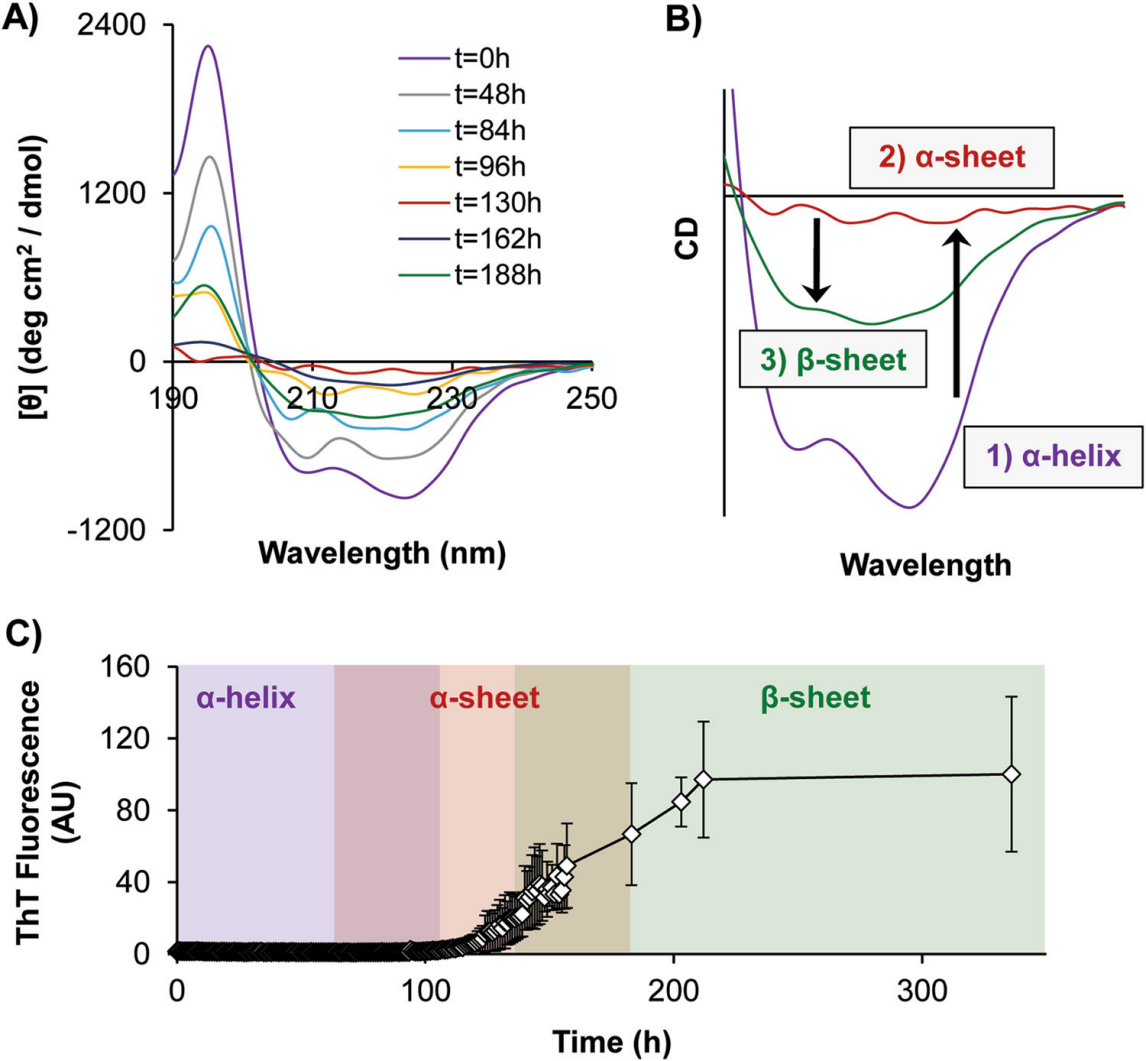
CD measurements capture structural transitions of PSMα1. **a** CD spectra of PSMα1 samples (30 μM, 1.3% HFIP, 50 mM potassium phosphate buffer, pH 5.5) were taken periodically during aggregation. At early time points (*t* = 0, 48, 84 h), negative peaks at ~208 and ~220 nm represent α-helical secondary structure. At intermediate time points (*t* = 130 h), featureless spectra indicate formation of α-sheet, and by the end of the time course (*t* = 188 h) a negative peak at ~218 nm signals the presence of β-structure. **b** Close-up view of characteristic CD spectra for α-helix (0 h, *purple*), α-sheet (*red*, 130 h), and β-sheet (*green*, 188 h). **c** Aggregation of synthetic PSMα1 peptide (30 μM, same conditions as for CD) was tracked over time by ThT fluorescence in a microtiter plate. Error bars in **c** represent the standard deviation of the mean of four samples

**Fig. 4:**
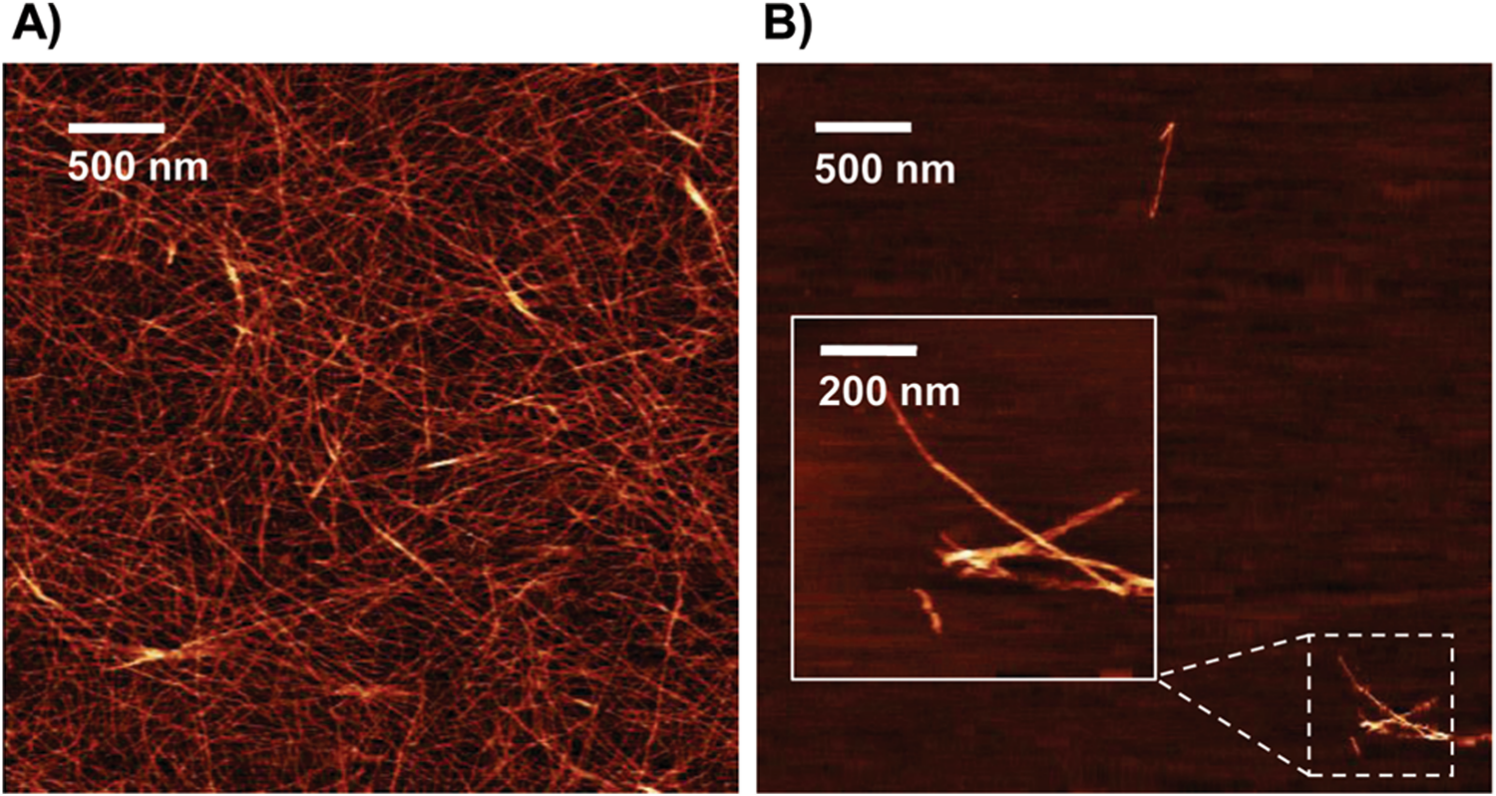
AFM images of synthetic PSMα1 amyloid fibrils. **a** PSMα1 peptide samples solubilized with DMSO were allowed to aggregate at high concentrations (437 μM), and the resulting fibrils exhibited extensive surface coverage, with each fibril measuring ~10 nm in diameter and 0.1–4 μM in length. **b** PSMα1 was also allowed to aggregate at 44 μM, yielding comparable but less dense fibrils

Solutions of HFIP-treated PSMα1 (30 μM, 1.3% v/v HFIP, potassium phosphate buffer, pH 5.5) were incubated at 37 °C and CD spectra were collected to determine the conformational species populated during aggregation (Fig. 3a and b). Simultaneously, aggregation was monitored in matched PSMα1 samples using ThT in a microtiter plate (Fig. 3c). At the beginning of the time course, the PSMα1 peptide displayed a characteristic α-helical spectrum by CD, with minima at ~208 and 222 nm. With time, however, the α-helical content decreased, as evidenced by the progressive loss of the helical signal through the first 100 h. As mentioned above, α-sheet gives rise to a featureless CD spectrum and the PSMα1 spectrum at 130 h is very similar to those of the designed α-sheet peptides (Supplementary Fig. S1). Prior to this time, there appeared to be progressive conversion of α-helix to α-sheet such that the mixing led to lifting of the helical signal until full conversion occurred. Notably, the “flattened” α-sheet spectrum preceded with the onset of fibril formation at ~130 h. As aggregation proceeded, a β-sheet CD signal appeared at ~217 nm. The onset of fibril formation (the length of the lag period) was inversely proportional to the concentration of the sample (Supplementary Fig. S3). As further confirmation of the presence of amyloid fibrils, PSMα1 was allowed to aggregate at different concentrations (shown for 437 and 44 µM in Fig. 4a and b, respectively) and the resulting samples were examined with Atomic Force Microscopy (AFM). Fibrils measuring ~10 nm in diameter and 0.1–4.0 μm in length formed at both concentrations (Fig. 4a and b). The mica substrates exhibited extensive surface coverage by a dense network of amyloid fibrils at high concentration.

### Designed α-sheet peptides inhibit amyloid formation through selective binding

**Fig. 5:**
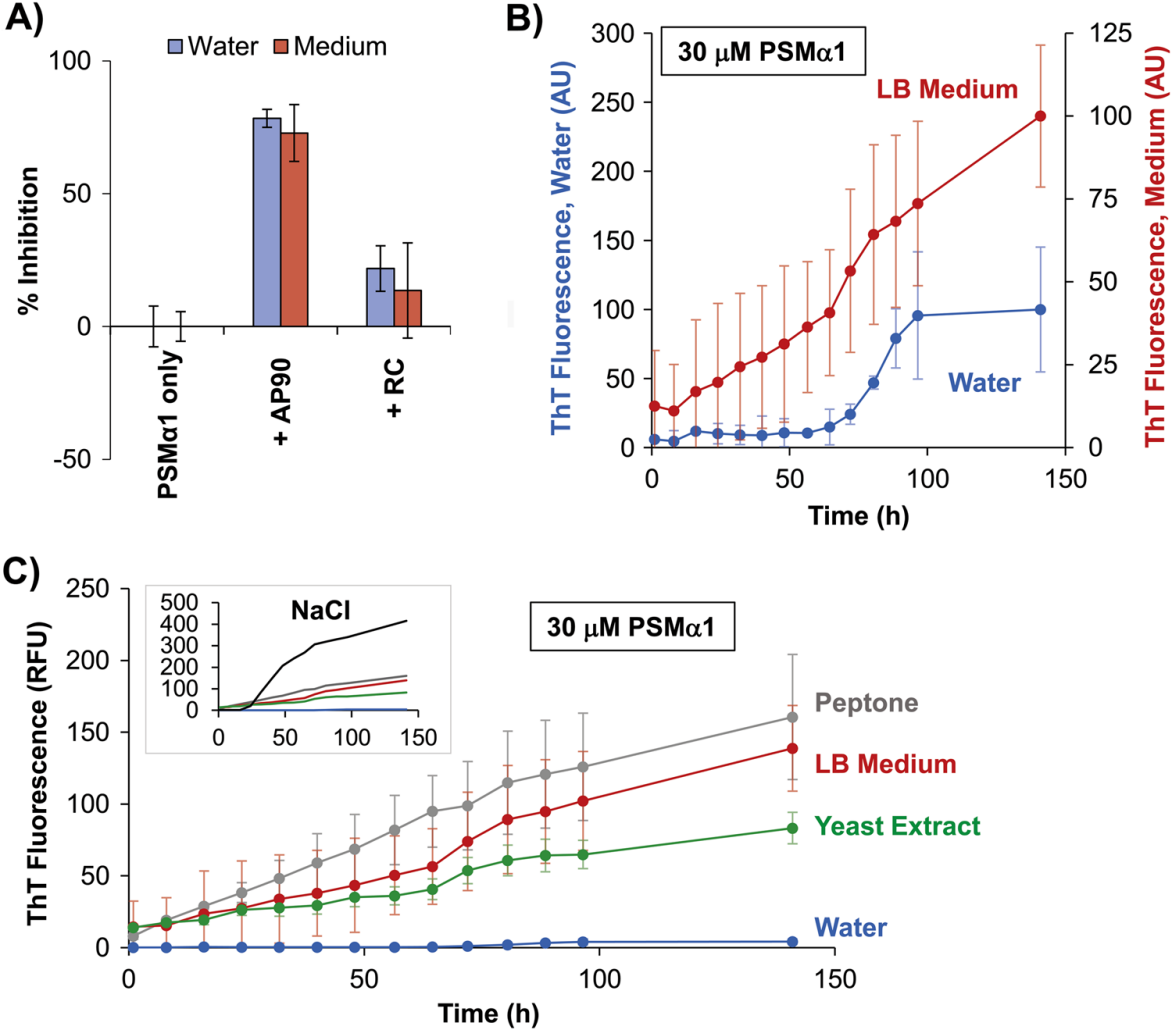
Aggregation of synthetic PSMα1 is inhibited by designed α-sheet peptides. **a** Synthetic PSMα1 peptide (30 μM, 0.34% DMSO, pH 5) was allowed to aggregate alone and in the presence of AP90 (1:4 molar ratio) and RC (1:4 molar ratio). Two different solvent conditions were used (0.34% DMSO in water, *blue bars*; LB medium + 0.34% DMSO, red bars) and aggregation was monitored by ThT fluorescence. Inhibition values for each peptide are reported as a percentage of the peptide-free control samples (0% inhibition). Error bars represent the standard error of the mean of 3–6 replicates. **b** ThT fluorescence curves monitoring the aggregation kinetics of PSMα1 under the two different solvent conditions in panel **a** (water = blue curve; LB medium = red curve). **c** The contribution of individual LB medium components (10 g/L peptone, 5 g/L yeast extract, and 85 mM NaCl) to PSMα1 aggregation kinetics was also investigated. Fluorescence values in NaCl solution were quite high, so its curve is shown as an inset. All solutions contain 0.34% DMSO for consistency and solubilization of PSMα1. Values in **b** and **c** are averages of 3 samples, corrected by relevant PSMα1-free controls, with error bars to represent the standard deviation of the mean and curves are corrected by subtracting blanks lacking PSMα1

After exploring the structural transitions involved in formation of amyloid in PSMα1, we examined the effect of the designed α-sheet peptides on the aggregation of PSMα1 in vitro. The designed peptide inhibitors were co-incubated with freshly prepared samples of synthetic PSMα1 pre-treated in DMSO (30 μM, 0.34% v/v DMSO, ddH_2_O, pH 5) and aggregation was monitored by ThT. Addition of AP90 at a 4:1 molar ratio inhibited PSMα1 amyloid formation by 81 ± 5% (Fig. 5a). The random coil peptide control had little effect.

Given the differences between the conditions of the in vitro aggregation reactions and those in the *S. aureus* cultures, we investigated the effect of LB medium on the behavior of PSMα1. As shown in Fig. 5b, the LB medium increased the rate of fibril formation, effectively abolishing the lag. In contrast, solubilization of PSMα1 in HFIP stabilized the α-helical structure, dramatically increasing the lag time (compare Figs. 3c and 5b). To further investigate the effect of the medium, aggregation of PSMα1 with the individual components of the medium was monitored (Fig. 5c). LB Lennox medium contains 85 mM NaCl, 10 g/L peptone, and 5 g/L yeast extract. The effect of the components was not additive; peptone increased the rate of aggregation of PSMα1 compared with medium containing the same concentration of peptone. In contrast, the yeast extract resulted in a slightly lower rate of aggregation compared to full LB medium. NaCl had little to no effect on the kinetics early on, but then the fluorescence increased dramatically (Fig. 5c, inset). The resulting signal with NaCl was extremely high and seems incompatible with the concentration of PSMα1, but it is attenuated in the presence of the other substituents in the LB medium. Despite the increased rate of aggregation of PSMα1 in LB medium, AP90 inhibited fibril formation to nearly the same extent as in water (73 ± 11%, Fig. 5a). Once again, addition of the RC peptide control displayed no inhibition.

**Fig. 6:**
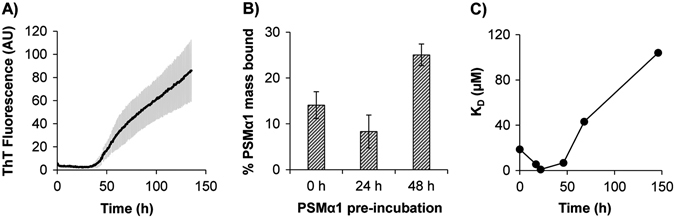
Designed α-sheet peptides preferentially bind α-sheet-rich PSMα1 over fresh or fibrillar PSMα1. **a** Synthetic PSMα1 peptides (30 μM, 0.34% DMSO, pH 5) were allowed to aggregate as in Fig. 5, and matched samples were removed periodically from the plate for binding assessment using an agarose bead, resin-based assay and biolayer interferometry. Error bars represent the standard deviation of six samples. **b** In the resin-based assay, AP193-functionalized beads preferentially bound α-sheet rich PSMα1 (48 h) over earlier time points (0 and 24 h). Error bars represent the standard error of the mean over six samples. **c** In biolayer interferometry experiments, the equilibrium dissociation constant, K_D_, indicates preferential binding between AP90 and α-sheet-rich PSMα1 (~48 h) as opposed to α-helix-rich (~0 h) or β-sheet-rich states (~150 h). Note that 150 h is still in the sigmoidal region of the transition and some α-sheet is present

To further probe the interactions between PSMα1 and the designed α-sheet peptides, we immobilized AP90 on agarose beads and applied solutions of either (a) fresh (completely solubilized), (b) pre-incubated, (allowed to aggregate as in Fig. 6a for 48 h corresponding to α-sheet oligomer species) or (c) fibrillar PSMα1. The bead mixtures were contained within a micro-spin column, so the system was treated as a packed bed reactor and a series of phosphate buffered saline (PBS) washes was used to remove unbound material. The PSMα1 content of each wash was measured with a fluorescence-based assay. PSMα1 bound to immobilized AP90 was eluted with high guanidinium hydrochloride (GndHCl). As can be seen in Supplementary Fig. S4, more of the oligomer sample bound to the column than fresh or fibrillar material. Unfortunately, however, the GndHCl needed to elute the bound PSMα1 obscures the fluorescence, leading to false positives, high error bars, and lack of confident confirmation of mass balance in the assay.

We then employed a hydrophobic AP90 derivative—termed AP193—in the agarose bead assay. The use of a slightly more hydrophobic peptide design enabled better coupling efficiency between the lysine and aldehyde functional groups on the bead surfaces (94% coupling efficiency with AP193 vs. 63% efficiency with AP90) while maintaining the desired α-sheet design structure.^25^ After functionalization with AP193, reactive sites on the beads were blocked, and fresh or pre-incubated PSMα1 samples were applied. Given the problem with GndHCl, the difference between the total amount of PSMα1 applied and that washed from the column (Supplementary Fig. S5) prior to elution of bound material with GndHCl was used to estimate of the mass of PSMα1 bound to AP193. According to CD (Fig. 3), we expect PSM α-sheet structures to become enriched near the end of the aggregation “lag phase”, which occurs between 45 and 50 h with DMSO solubilized material (Fig. 6a). As shown in Fig. 6b, pre-incubated PSMα1 (48 h) was preferentially bound over helical PSMα1 (0 and 24 h).

The binding was explored further using biolayer interferometry, which analyzes interference patterns based on adsorption of samples to a biosensor tip. For this study, AP90 was immobilized on the tip, and then the association and dissociation of PSMα1 was measured at various time points during aggregation. In agreement with the agarose bead-binding experiments, PSMα1 samples near the end of the lag phase displayed the highest binding affinity (*K*_D_ = 0.9–6.8 μM, Fig. 6b and c), and the binding affinity dropped by two orders of magnitude to 104 μM as PSMα1 converted to β-sheet (~150 h).

## Discussion

PSMs play significant and varied roles in the biofilm matrix in *S. aureus*. In their soluble, α-helical form, these small peptides act as surfactants to disperse the biofilm and promote downstream colonization.^14^ On the other hand, PSMs can aggregate to form functional amyloid fibrils that stabilize biofilms and provide resistance to disruption,^15^ which is critical to the virulence of medical device-associated infections. Through these two modes of action, *S. aureus* communities take advantage of the unique properties of PSMs to thrive under a variety of conditions within the host. The prevalence of PSMs in drug-resistant infections,^29^ combined with their ability to influence biofilm development, has made them an attractive target for therapeutic intervention in recent years.^30^ However, there have been no efforts to date to inhibit PSM amyloid formation. In this study, we have demonstrated a novel approach to suppress amyloidogenesis in the *S. aureus* biofilm matrix through the use of *de novo* α-sheet peptides.

The designed α-sheet peptides inhibited amyloid formation in both a laboratory strain of *S. aureus* (SH1000 WT), as well as a human urogenital isolate (MN8), and the effect was dose-dependent. In fact, all three of the α-sheet designs inhibited fibril formation, while the random coil and β-hairpin control peptides did not. Furthermore, inhibition was accompanied by a weakening of biofilm attachment or the biofilm matrix, presumably induced through targeting of PSM aggregation and assembly of the fibrillar scaffold.^15^ Interestingly, while ThT fluorescence indicated that the AP90 design only inhibited amyloid formation by ~30% in *S. aureus* SH1000 biofilms, no amyloid fibrils were visible when these biofilms were imaged by TEM. This suggests that ThT may bind to other species in addition to the amyloid fibrils or that smaller ThT-binding protofibrils may be present but not visible in the TEM images.

As a simple bridge between the inhibition studies in cells and with pure PSMα1, aggregation of synthetic PSMα1 was investigated in two different solvent conditions—water and LB medium. The lag phase was eliminated in the LB medium, suggesting that components of the growth medium accelerated aggregation. In addition, components of the medium gave rise to higher fluorescence even after correcting for their high fluorescence in the absence of PSMα1. This presents a variety of possibilities including that LB components interact with the fibrils and bind or trap ThT, that they differentially bind ThT when PSMα1 is present, or that they affect the fluorescence yield. Interestingly, however, investigation of the individual media components shows that there are compensating ‘buffering’ interactions in fully formulated LB media. Nonetheless, despite the inclusion of both known and unknown components, the inhibition by AP90 persisted. These observations reinforce our hypothesis that specific interactions between PSMs and designed α-sheet inhibitors lead to a reduction of amyloid fibril content in *S. aureus* biofilm cultures. It is possible that other staphylococcal matrix components may also accelerate aggregation in vivo, as shown for extracellular DNA,^31^ but they are not required for amyloid formation.

To better map the process of conversion, PSMα1 conformational behavior was investigated in aqueous solution. Independent of PSMα1 concentration, the aggregation reactions ultimately resulted in fibrils ~10 nm in diameter and 0.1–4.0 μm in length (Fig. 4). These are similar to mammalian amyloid fibrils, which typically also span 10 nm in diameter (with a range of 5–25 nm) and up to 10 μm in length.^32^ The AFM images of PSM fibrils confirm that their size and morphology are indeed amyloid-like. Given that mature fibrils contain cross-β-structure, it is often assumed that intermediate species generated on the pathway to amyloid formation must also contain β-structure. However, it has been shown in several amyloid systems that these intermediate species lack β-structure.^33–35^ These studies are in agreement with our CD results for PSMα1, as it converted from soluble monomers to insoluble fibrils, progressing from α-helix→α-sheet→β-sheet fibrils. Correspondingly, our designed anti-α-sheet peptides, which are themselves α-sheet hairpins, target the intermediate α-sheet structure of PSMα1 and inhibit amyloid fibril formation in vitro, supporting a recent study showing that PSMα1 has a high aggregation propensity compared to the other α-PSMs.^36^ The conformational equilibria are complicated, but we observed entry and exit from α-sheet occurring just prior to the onset of a steep increase in ThT binding, with mixed populations before and after with α-helix and β-sheet, respectively. The end of the lag phase of aggregation was also associated with preferential binding of α-sheet-rich PSMα1 samples to beads functionalized with AP193, as well as the increased binding affinity observed by biolayer interferometry.

Interestingly, a crystal structure of PSMα3 fibrils was just reported composed of stacked α-helices, not β-sheets.^37^ This challenges the long-held dogma that amyloid is necessarily cross β-sheet. While fascinating, it’s not clear how general this finding is for PSM peptides in particular and amyloid proteins in general. PSMα1 and PSMα3 have low sequence identity (7 residues in common). PSMα1 undergoes a transition from α-helix→α-sheet→β-sheet during aggregation as determined by matched aggregation time courses and CD measurements. Unfortunately the CD was not evaluated as a function of time in the PSMα3 study.^37^ Others have noted α-helical supernatant and β-sheet pellets within a single sample,^15^ which could confound structural studies. Given the complicated conformational equilibria and sensitivity to concentration, incubation time and solution conditions, as well as the lack of continuity in the conditions and timing of the various experiments, making direct links between structure, aggregation and toxicity is difficult. PSMα3 is one of the most toxic PSMs and small amounts of PSMα3 have been detected in 10 different community-acquired and hospital-acquired MRSA strains.^38^ However, PSMα3 has not been detected in *S. aureus* fibrils while the existence of PSMα1, PSMα2, and PSMα4 has been well documented in *S. aureus* amyloid fibrils.^15^

The functional amyloid of *S. aureus* represents an interesting and complicated system with many components and opportunities for regulation, and further research into the details of individual components as well as more holistic approaches are warranted. Together, the CD, column-binding, inhibition assays and biolayer interferometry experiments presented here reinforce our hypotheses that amyloid formation by PSMα1 is characterized by formation of α-sheet structures and that the interaction between designed α-sheet peptides and PSMα1 is structure-specific. Thus, the present study lays the foundation for next-generation peptide designs to reduce the amyloid content of the biofilm matrix, rendering the biofilms less robust. Additionally, by removing the structural reinforcement of the biofilm EM provided by amyloid fibrils, these peptides may help mitigate problems with antibiotic transport and associated resistance.^39^

## Conclusions

The results presented here support our hypothesis that α-sheet is a generic structure formed during amyloidogenesis independent of source, sequence and structure. We have previously shown that *de novo*, designed α-sheet peptides inhibit aggregation of Aβ, amylin, and transthyretin, which are all human but unrelated by sequence and structure.^25, 26^ Here we provide further support for the generality of the α-sheet hypothesis in amyloidogenesis by showing that the assembly of a bacterial amyloid is also inhibited by these same designed α-sheet peptides and that the main proteinaceous component of the *S. aureus* amyloid fibrils, PSMα1, adopts α-sheet, supporting our inhibitor design premise to provide a complementary α-sheet surface. Thus, our design approach may be applicable to a variety of different functional bacterial amyloid systems and could represent a new class of therapeutic agents for biofilm-associated infections.

## Materials and methods

### Computational peptide design and synthesis

The computational design process has been described previously,^25^ but it is briefly outlined here. Our goal is stable, soluble α-sheet hairpin peptide designs (α-strand—turn—α-strand to form a small α-sheet hairpin). To do this we designed a turn to support the necessary geometrical requirements of α-sheet and strands comprised of alternating L/D amino acid sequences, as L-amino acids favor α_R_ conformations and the corresponding D-amino acids favor α_L_ conformations.^40, 41^ In addition, amino acids were chosen to favor particular packing across the strands, as well as for solubility. For this study we focused on variants to our benchmark AP90 design: AP401, reversed chirality with respect to AP90; AP407, contains a disulfide bond near the turn; and AP193, a variant to support different chemical coupling reactions (Supplementary Table S1). The peptides were produced using solid phase peptide synthesis on Rink amide resin with Fmoc chemistry and HBTU activation.^42^ The resulting resin-bound peptides were cleaved and side chains deprotected with (trifluoroacetic acid) TFA/TIPS/H_2_O (95:2.5:2.5) and precipitated with cold ether. Crude peptides were purified by RP-HPLC to ~98% purity (Phenomenex 5 μm C12 100 Å semiprep column). Purified peptides were confirmed by mass spectrometry (MS) on a Bruker Esquire Ion Trap electrospray mass spectrometer, and peptide stocks were lyophilized for storage at −20 °C. For assays, peptide stocks were thawed and reconstituted in filtered ddH_2_O to a concentration of 2 mg/mL.

### Biofilm screening assays

Overnight cultures of *S. aureus* (see Supplementary Table S2 for a list of strains) were spun down and re-suspended in fresh LB (Lennox) medium to an optical density of 0.1 (600 nm). These cultures were then mixed with reconstituted peptide (or water, in the case of controls) and aliquoted in quadruplicate into wells of a clear 48-well plate (Corning, TC-treated polystyrene). The final concentration of peptide in each well was 80 μM, unless otherwise noted. Plates were covered and biofilms were grown at 37 °C for 24 h with gentle rocking. Medium and planktonic cells were then removed from wells using a vacuum and the remaining adherent biofilms were rinsed once with PBS (BupH, Thermo Scientific).

ThT, an established fluorescent label for amyloid fibrils,^43^ was then added to each well at a concentration of 22 μM and biofilms were incubated statically for 4 h at room temperature. The solution was then removed from the wells and PBS was added with vigorous pipetting to detach biofilms. Plates were shaken at high speed for 1 min on a plate shaker to detach any remaining biofilm material and homogenize the samples, which were then transferred to a 96-well black-walled plate (Corning, TC-treated polystyrene) and ThT fluorescence was measured using an excitation wavelength of 438 nm and emission at 495 nm on a Perkin–Elmer Enspire plate reader (hereafter referred to as the plate reader). Fluorescence measurements were corrected by subtracting the background intensity of identical samples without ThT (in the case of the biofilms) or PSMα1 (for the in vitro measurements). In the case of Crystal Violet staining, biofilms were grown and rinsed with PBS in the same manner as the ThT assay. After rinsing, a solution of 0.5% w/v Crystal Violet dye was added to each well and biofilms were stained for 15 min. Excess stain was removed and biofilms were allowed to dry overnight prior to addition of 30% v/v acetic acid to solubilize the biofilms. Solubilization was allowed to proceed for 15 min at room temperature, and then the stained solutions were transferred to a new microtiter plate for measurement of absorbance at 550 nm on the plate reader.

### Microscopy

For fluorescence microscopy studies, *S. aureus* MN8 + *mCherry* overnight cultures were diluted to an optical density of 0.1 (600 nm) and combined with α-sheet inhibitors as described above; the final concentration of peptide was 80 μM. Biofilms were grown in sixteen-well chambered coverglass plates (Lab-Tek^®^ Chamber Slides) and analyzed on a Zeiss Axio Observer inverted microscope after gentle washing and fixing with 4% paraformaldehyde. For each condition, 4–6 images (field size = 0.074 mm^2^) were collected. Cell counts were performed in ImageJ by adjusting the color threshold and then using the particle analysis feature.

For AFM studies, the synthetic PSMα1 peptide was prepared to a concentration of 44 and 437 μM (as described below, DMSO solubilization) and incubated at 37 °C. The resulting amyloid fibrils were applied directly to freshly cleaved mica and incubated for 2 h. Samples were rinsed five times with ddH_2_O and allowed to dry prior to imaging on a Bruker ICON atomic force microscope using tapping mode and a ScanAsyst silicon tip. Images were analyzed using Gwyddion software (Czech Metrology Institute). For TEM, biofilms were grown in 48-well plates as described above. After 24 h of growth, biofilms were rinsed with PBS, scraped from the sides of the plate, spotted onto formvar-coated copper grids, stained with 2% uranyl acetate for 2 min, and imaged on a JEOL-1230 microscope with an AMT XR80 camera.

### Preparation of PSMα1 peptide

Lyophilized peptide stocks of synthetic PSMα1 (fMGIIAGIIKVIKSLIEQFTGK, where f denotes formylation, Ontores Biotechnologies) were prepared as described previously^15^ to eliminate aggregates from lyophilization prior to assay. Briefly, dry PSMα1 peptide was dissolved to a concentration of 10 mg/mL in a 1:1 mixture of TFA and HFIP. Ice-cold HFIP was then added to dilute PSMs to 1 mg/mL and the sample was sonicated for 10 min. Solvent TFA/HFIP was removed by air stream and then speedvac at room temperature before storage at −20 °C. Prior to assay, PSMα1 aliquots were subjected to a secondary HFIP treatment. The peptide was dissolved in ice cold HFIP to a concentration of 10 mg/mL, vortexed, sonicated for 5 min, and incubated for 25 min on ice. The peptide was then dried to a film using a stream of air and vacuum (Savant SpeedVac concentrator) at room temperature. PSMα1 is very hydrophobic and requires a small amount of organic solvent for solubilization. At this point either DMSO or HFIP was utilized to dissolve the peptide film. DMSO was used for the bulk of the experiments but it has strong UV absorption, precluding its use for CD.

### CD spectroscopy

An HFIP-treated film of PSMα1 (0.1 mg) was solubilized with HFIP (20 μL) and diluted to 30 μM in potassium phosphate buffer (50 mM KH_2_PO_4_, pH 5.5) with and without 22 μM ThT, resulting in 1.3% v/v HFIP in the diluted samples. To determine the timing of CD measurements, PSMα1 polymerization was monitored over time. 150 μL samples with and without ThT were aliquoted into individual wells of a black-walled 96-well plate (Corning) and the plate was incubated at 37 °C inside the plate reader. ThT fluorescence was measured every hour after shaking. ThT-free samples were periodically withdrawn from the plate for CD measurements at 37 °C on a Jasco J-715 spectrophotometer with 1 mm cuvettes. All spectra were smoothed (Savitsky–Golay method, convolution width 25, polynomial order 2) and deconvoluted (FWHM 10–15 cm^−1^) using Jasco Spectra Analysis software.

### PSMα1 fibrillization assay with added inhibitors

To solubilize the PSMs for the aggregation assay, filtered DMSO was added to a treated film of PSMα1 to achieve a 20 mg/mL solution. Samples were then further diluted by addition of 22 μM ThT in ddH_2_O or LB medium/components with and without added α-sheet peptide designs (at 1:4 molar ratio of PSMα1: inhibitor) resulting in a final concentration of 30 μM PSMα1, 22 μM ThT, and 0.34% DMSO in all cases. The LB medium (Lennox) components were formulated as three separate solutions: 10 g/L peptone, 5 g/L yeast extract, and 85 mM NaCl. Fifty microlitre samples were aliquoted into 384-well black-walled plates (BrandTech, nontreated polystyrene). The plate was incubated in the plate reader at 37 °C and ThT fluorescence was measured every 30–60 min.

### Immobilization and solution binding

Peptide designs were immobilized on Pierce Amino Link agarose beads in a Pierce spin column (Thermo Fisher Scientific).^25^ Briefly, the designed peptide AP193 (Supplementary Table S1) was dissolved to a concentration of 250 µM in PBS + 25% v/v DMSO + 50 mM NaCNBH_3_ and allowed to couple to the aldehyde-functionalized resin overnight at 4 °C. Residual active sites were blocked with 1 M Tris HCl + 50 mM Na CNBH_3_ for 4 h at 25 °C. Meanwhile, PSMα1 samples were prepared as above (see “PSM fibrillization assay”) and incubated at 37 °C. After 0, 24, and 48 h, a sample of 200 µL PSMα1 was removed from the microtiter plate and added to a prepared spin column, where the samples were allowed to bind to the peptide-functionalized resin beads for 2 h at 25 °C. The solution was then collected by centrifugation (flow-through, FT). The beads were re-suspended in 300 µL PBS, vortexed to obtain a uniform slurry, and then the solution was collected by centrifugation (wash 1, W1). This wash step was performed a total of seven times, until no remaining protein was detected in the eluent (Supplementary Fig. S5). FT and the washes (W1-W7) were analyzed with the NanoOrange^®^ Protein Quantitation Kit (Thermo Fisher Scientific). The masses of the eluents were summed and subtracted from the mass of PSMα1 applied to the column, providing a rough estimate of the bound material. This approach was necessary because elution of bound PSMα1 with GndHCl obscures protein quantification, and unfortunately, the inability to quantify the GndHCl bound fractions prevents confirmation of the mass balance.

### Biolayer Interferometry

All biolayer interferometry experiments were performed on a BLItz biosensor system (ForteBio, Pall) using aminopropylsilane (APS) sensors. Sensors were hydrated in ddH_2_O + 22 μM ThT for 10 min prior to use; ThT was included to mirror the conditions of the corresponding aggregation curves. AP90 (180 μM dissolved in ddH_2_O + 22 μM ThT) was loaded onto the APS tip, a baseline was established in ddH_2_O + 22 μM ThT, and then the association of PSMα1 (50 μM prepped with DMSO + ddH_2_O + 22 μM ThT, as in the fibrillization assay) at various pre-incubation times was monitored over a period of 3 min. Dissociation was subsequently measured in ddH_2_O + 22 μM ThT, and the equilibrium dissociation constant (K_D_) was calculated using the BLItz analysis software (ForteBio, Pall).

### Data availability

Data generated and analyzed during this study are included in this published article and its supplementary information file.

## Electronic supplementary material

SI
