# Supplementary material for: Designed α-sheet peptides suppress amyloid formation in Staphylococcus aureus biofilms
Source: NPJ Biofilms Microbiomes. 2017 Jul 3;3:16. doi: 10.1038/s41522-017-0025-2 (PMC5495782; doi:10.1038/s41522-017-0025-2)
Supplement: Supplementary file 1 — SI [file 41522_2017_25_MOESM1_ESM.pdf]

Supplementary information for: Designed α-sheet peptides suppress amyloid formation in *Staphylococcus aureus* biofilms

Alissa Bleem, Robyn Francisco, James D. Bryers, and Valerie Daggett[*]

Department of Bioengineering, University of Washington, Seattle, Washington, 98195-5013, USA

***Correspondence to:** Valerie Daggett; daggett@uw.edu

Table S1. Peptide designs used in this study

All designs are single-turn α- or β-sheet hairpins, with the exception of *RC* (random coil). The peptides are N- and C-terminally acetylated and amidated, respectively, except for *β*, which has a free N-terminus. Lower case and underlined letters denote D-amino acids, turn residues are shown in red, and disulfide-bonded cysteines in AP407 are shown in bold.

Controls	*RC*	Ac-KLKpLLTSENTL-NH_2
	β	SWTWEpNKWTWK-NH_2
α-sheet Designs	*AP90*	Ac-RGEmNlSwMNEYSGWtMnLkMGR-NH_2
	AP401	Ac-rGeMnLsWmneysGwTmNlKmGr-NH_2
	AP407	Ac-RGEmNl**C**wMNEYSGW**c**MnLkMGR-NH_2
	AP193	Ac-RGEmNyFwMNEYYGWtMnCkMGR-NH2

Table S2. Bacterial strains used in this study

Strain	***Description***
S. aureus SH1000 (WT)	Laboratory strain, *rsbU*$^+$
S. aureus MN8	Clinically relevant strain; urogenital tract
S. aureus MN8 + *mCherry*	Transcriptional fusion of RFP to the P3 promoter of strain MN8

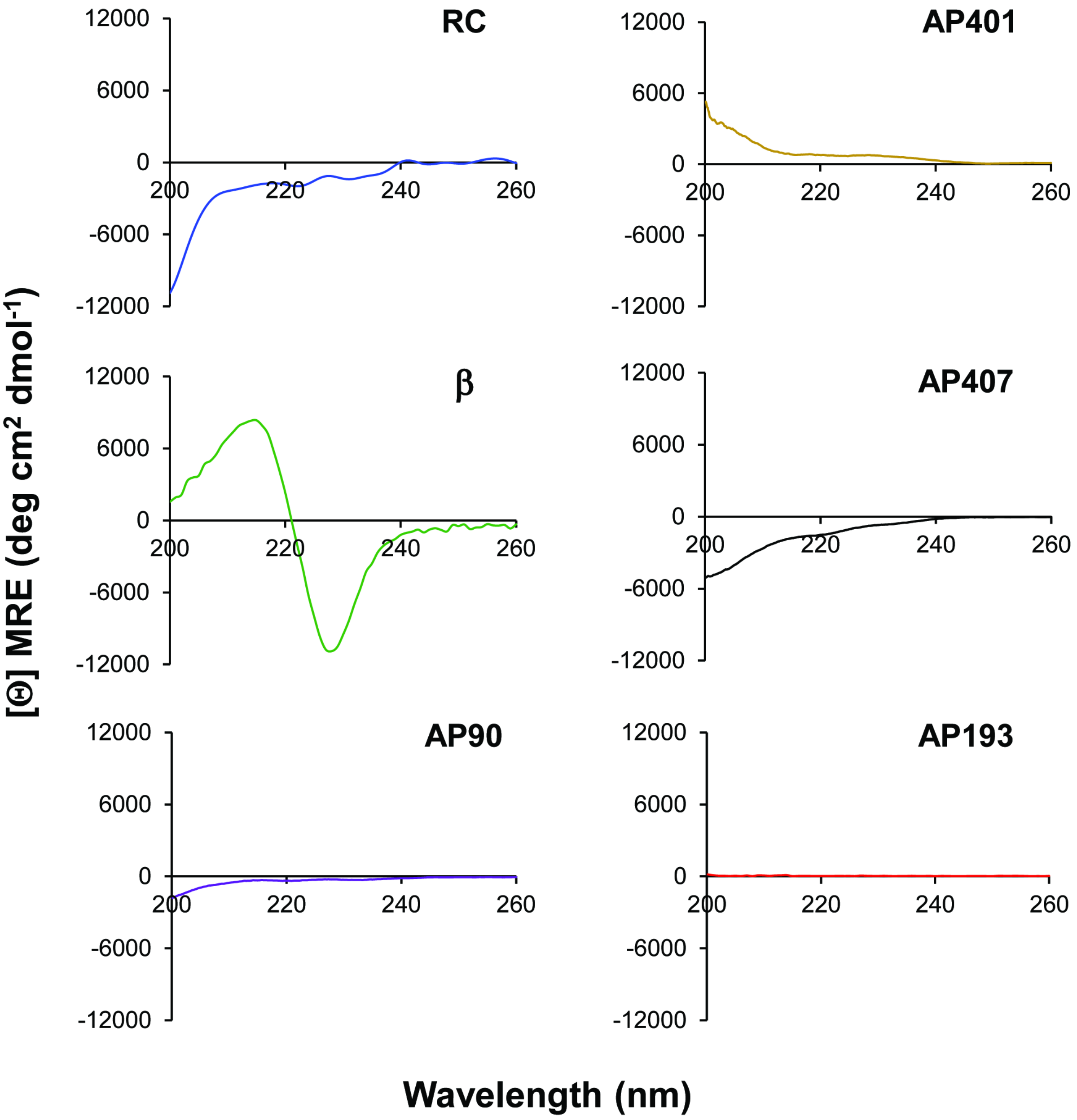

Fig S1. CD spectra of RC, β, AP90, AP401, AP407, and AP193. Peptides were dissolved to a concentration of 100 μM (except for RC, which was 30 μM) in phosphate buffer (except for β and AP193, which were in acetate buffer) and spectra were acquired at room temperature. [Θ] MRE = mean residue ellipticity. Note that the β-sheet spectrum of *β* is obscured by the Cotton effect manifested by the interaction of W-W residues across the sheet, and further structural information for this peptide has been presented elsewhere (Cochran et al., Tryptophan zippers: stable, monomeric β–hairpins. Proc. Natl. Acad. Sci. U.S.A. 2001, 98, 5578–5583).

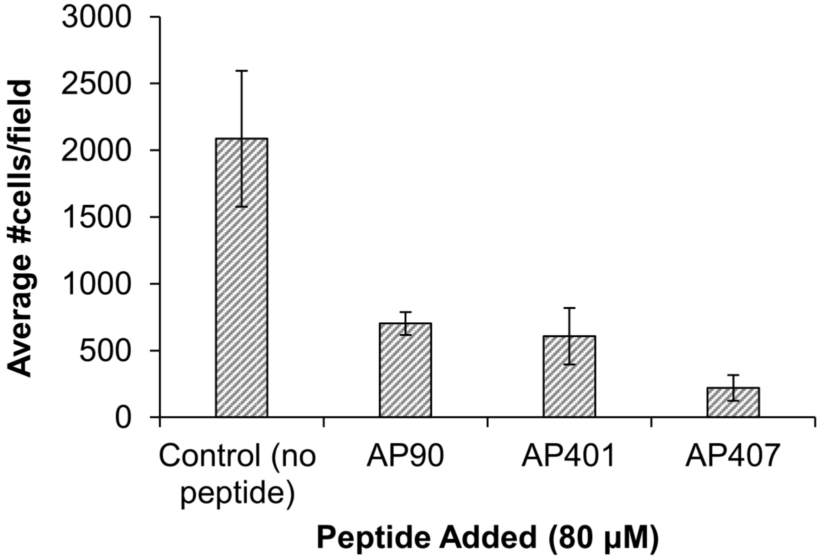

Fig. S2. Designed peptides reduce the number of cells attached to glass slides after washing. The average number of cells per field was determined for each image collected and averaged over triplicate images. Error bars represent the standard deviation of the mean. This figure goes with the images in Figure 2.

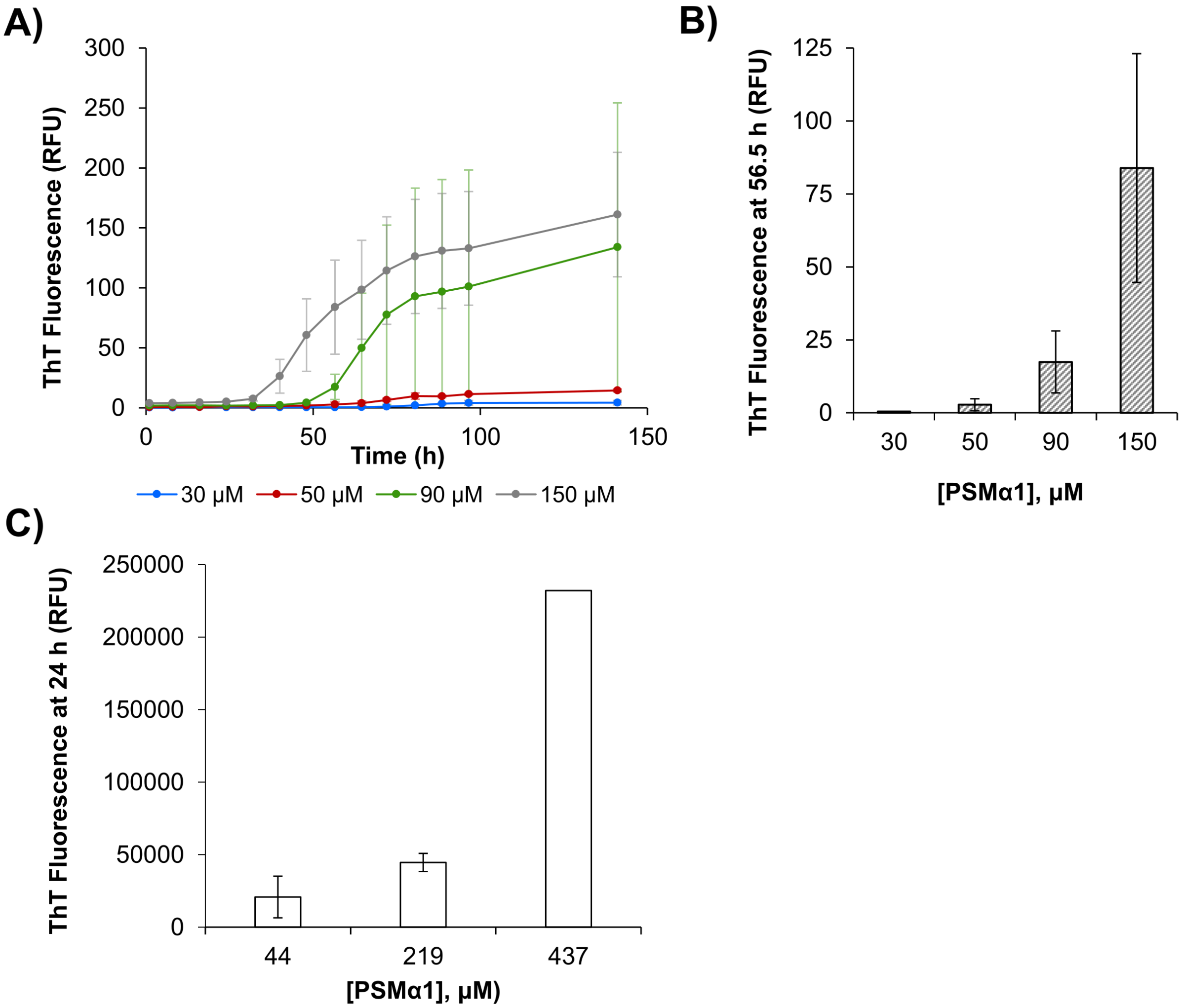

Fig. S3. The rate and extent of amyloid formation by synthetic PSMα1 depends on peptide concentration. **(A)** Synthetic PSMα1 peptide (0.34% DMSO in water, pH 5.5) was allowed to aggregate at four different concentrations (30, 50, 90, and 150 μm) and amyloid formation was monitored by ThT fluorescence. Higher concentrations of PSMα1 resulted in faster rates amyloid formation. **(B)** ThT fluorescence measurements from panel A shown for 56.5 h, which indicates there was greater extent of amyloid formation for higher concentrations of PSMα1. **(C)** In the case where PSMα1 was allowed to aggregate for AFM experiments, a similar correspondence between ThT fluorescence values and PSMα1 concentration was observed. Experiments were performed in triplicate, and error bars indicate the standard deviation from the mean.

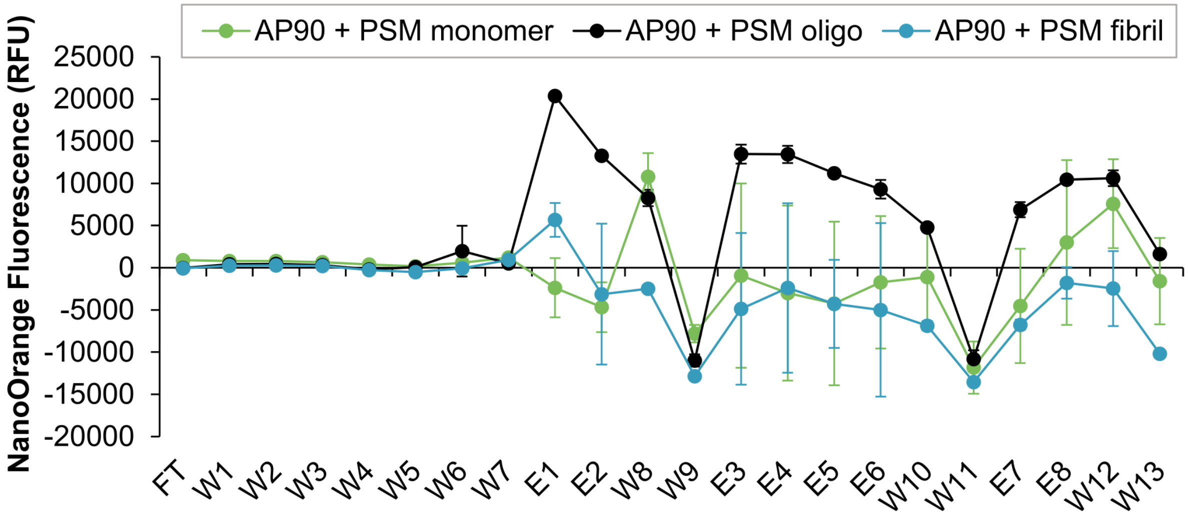

Fig. S4. The designed peptide AP90 was dissolved to a concentration of 250 μM in PBS + 50 mM $NaCNBH_3$ and immobilized on Pierce Amino Link agarose beads in a Pierce spin column (Thermo Fisher Scientific) overnight at 4°C. Residual active sites were blocked with 1 M Tris HCl + 50 mM $NaCNBH_3$ for 4 h at 25°C. Meanwhile, PSMα1 samples were prepared as in the "PSM fibrillization assay" (Figure 6A) and incubated at 37°C. Solutions of either fresh (0 h), pre-incubated (allowed to aggregate for 48 h corresponding to α-sheet oligomer species), or fibrillar (165 h) PSMα1 were added to the prepared spin columns and allowed to bind to the peptide-functionalized resin beads for 2 h at 25°C. The solution was then collected by centrifugation (flow-through, FT). The beads were re-suspended in 300 μL PBS, vortexed to obtain a uniform slurry, and then the solution was collected by centrifugation (washes, W1-W7). GndHCl was then added to the columns at three different concentrations (E1-E2, 2 M GndHCL; E3-E6, 4 M GndHCl; E7-E8, 6 M GndHCl) to elute bound PSMα1. The GndHCl elution steps were alternated with PBS washes (W8-W13). All eluents were then analyzed with the NanoOrange® Protein Quantitation Kit and fluorescence was used as an indicator of PSMα1 content in each eluent. Fluorescence measurements were normalized by the corresponding protein-free (no PSMα1 added) eluents from a blank (active sites blocked but no AP90) spin column. Error bars represent the standard error of the mean over six samples.

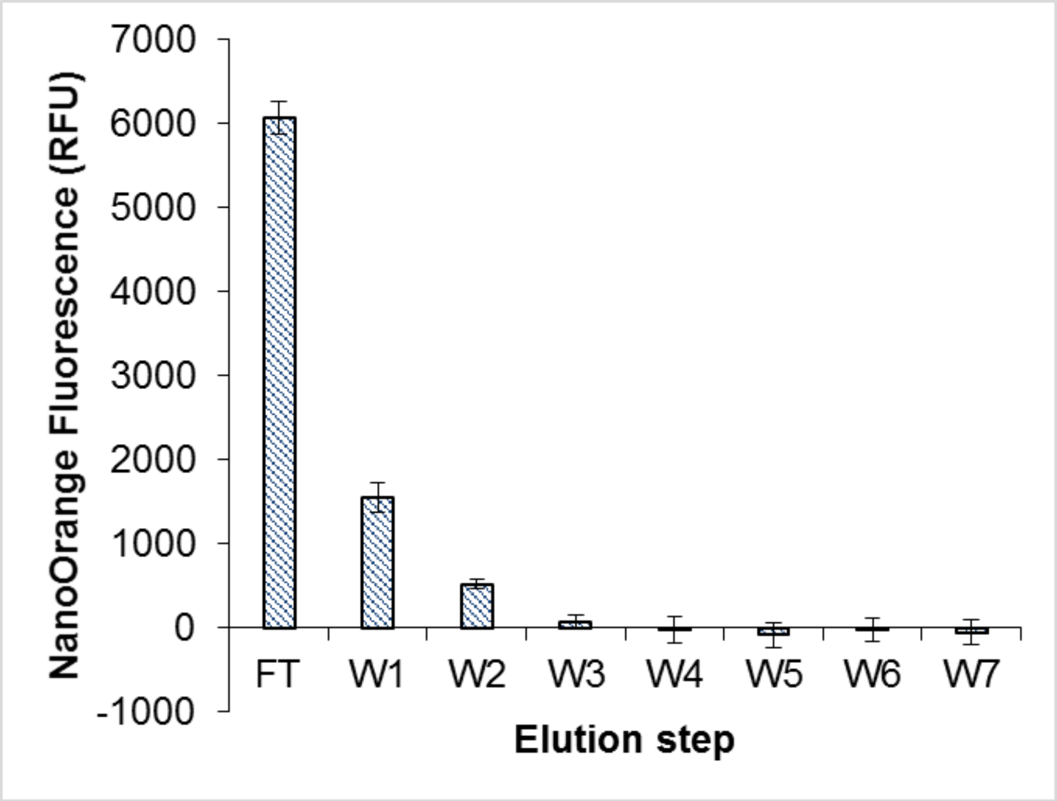

Fig. S5. Wash steps in the column-binding assay utilizing bound AP193 are sufficient to remove all detectable protein in the eluent. By the seventh wash, protein concentrations fall below the fluorescent-detectable level of 10 ng/mL. The data shown here are for fresh PSMα1 (0 h) samples applied to the column, but they are representative of all column-binding assays. Error bars represent the standard error of the mean over six samples.
